# Reviewed article: Junior CAR, Santos SS, Amaral TLM. Trend and burden of lower-limb amputations due to diabetes: time series analysis, Amazonas, 2012-2021. Epidemiol Serv Saude. 2026:35;e20250534

**DOI:** 10.1590/S2237-96222026v35e20250534.b

**Published:** 2026-03-16

**Authors:** Mariana Del Grossi Moura

**Affiliations:** 1 Universidade de Sorocaba, Sorocaba, SP, Brazil Sorocaba SP Brazil

## First round comments

Agradeço a submissão do manuscrito RESS-2025-0534 intitulado “Tendência e carga de amputações de membros inferiores por diabetes no Amazonas, Brasil: análise de série temporal, 2012-2021” à Revista Epidemiologia e Serviços de Saúde: revista do SUS (RESS).

Após a revisão do seu artigo, identificamos a necessidade de uma revisão maior para que o manuscrito esteja de acordo com os padrões editoriais da revista e para viabilizar sua publicação.

Além dos pontos identificados pelos revisores, recomendo atenção:

- Os resultados apresentados baseiam-se no número de procedimentos de amputação registrados no SIH/SUS, e não no número de pessoas distintas submetidas a esses procedimentos. Essa limitação pode levar à superestimação das taxas de amputação e da carga da doença (YLD), especialmente nos casos em que uma mesma pessoa tenha sido submetida a mais de uma amputação ao longo do período analisado. Por esse motivo, recomenda-se que essa limitação seja descrita de forma clara na seção de métodos e discutida de maneira adequada nos resultados e na discussão, incluindo suas possíveis implicações na interpretação das diferenças observadas entre os sexos e entre as faixas etárias.

- Explicitar como foi definida a duração da incapacidade (D) no cálculo do YLD. Esclarecer se foi considerada como permanente, ou seja, até o final da expectativa de vida do indivíduo, e indicar qual fonte foi usada para essa estimativa. Essa informação é importante porque influencia diretamente o valor final do YLD.

- Justificar a escolha da estratificação geográfica por regiões intermediárias, esclarecendo se a localização dos casos foi definida com base no município de residência dos pacientes ou no local de realização do procedimento. Considerando a centralização dos serviços de média e alta complexidade em municípios-polo, como Manaus, é importante discutir como essa concentração pode ter influenciado a distribuição espacial observada dos procedimentos e os resultados apresentados.

Todos os comentários da revisão por pares devem ser respondidos pontualmente por meio de uma carta resposta detalhada, explicando as modificações realizadas ou justificando, quando necessário, a manutenção do texto original.

A nova versão do manuscrito será reconsiderada após essa reformulação e poderá passar por nova rodada de avaliação.

Recommendation: Major revision

## Second round comments

Agradeço a submissão do manuscrito RESS-2025-0534.R1 intitulado “Tendência e carga de amputações de membros inferiores por diabetes: análise de série temporal, Amazonas, 2012-2021” à Revista Epidemiologia e Serviços de Saúde: revista do SUS (RESS).

Após a revisão do seu artigo, identificamos a necessidade de uma revisão menor para que o manuscrito esteja de acordo com os padrões editoriais da revista e para viabilizar sua publicação.

Observações:

- Resumo: No trecho “A carga da doença totalizou mais de 21 mil anos vividos com incapacidade. Os homens (70,4%) apresentaram uma carga superior à das mulheres (29,6%)”, ajustar de forma a deixar claro que os percentuais apresentados correspondem à proporção da carga total de anos vividos com incapacidade atribuída a cada sexo; utilizar o valor exato obtido no estudo, em lugar da expressão genérica “mais de 21 mil”, para garantir precisão e objetividade; reescrever a conclusão de modo a limitar-se estritamente aos resultados observados, sem extrapolar para “vulnerabilidade”.

- Aspectos éticos: A seção deve ser removida do corpo do manuscrito, permanecendo apenas na folha de rosto

- Método: revisar a seção “Variáveis e fonte de dados” para garantir alinhamento completo entre o método e as categorias apresentadas nas tabelas/resultados.

- Resultados: (i) uniformizar a terminologia das faixas etárias entre texto e tabelas, substituindo “65 anos e mais” por “≥65 anos”; (ii) ajustar a expressão “79,23 YLD/100 mil habitantes ano” para “79,23 YLD por 100 mil habitantes/ano”, a fim de evitar ambiguidades; (iii) reforçar, ao mencionar os “1.749 casos incidentes”, que se trata de estimativa ajustada pela média de reamputações, para maior transparência; e (iv) revisar trechos como “a maior parte do YLD se concentrando” para uma forma no passado, em concordância com os demais achados (ex.: “a maior parte do YLD concentrou-se na faixa etária de 45 a 64 anos”)

- Discussão: a seção está muito longa, com informações repetidas ou de pouco valor agregado (ex.: detalhamento excessivo de tendências internacionais). É recomendável condensar, mantendo foco nos achados do estudo e suas implicações para o Amazonas; alguns termos extrapolam os resultados. O uso de “maior vulnerabilidade masculina” não é adequado, já que o estudo mostra apenas maior carga e tendência em homens, sem avaliar determinantes sociais ou culturais. Sugere-se substituir por “observou-se maior carga e tendência crescente em homens”; Reformular a conclusão para que seja curta, clara e estritamente alinhada ao objetivo e resultados do estudo.

- Disponibilidade dos dados: deve ser informado onde os leitores podem encontrar o banco analisado na presente pesquisa. Não se trata da fonte de dados, mas o banco de dados utilizado para análise, bem como códigos e demais dados pertinentes para assegurar transparência na pesquisa. Favor observar a política editorial da RESS, item Dados abertos: "Os dados de pesquisa (bancos de dados, códigos, métodos e outros materiais utilizados e resultantes da pesquisa) idealmente devem ser depositados em repositórios de dados confiáveis como o repositório SciELO Data (lista disponível em https://wp.scielo.org/wp-content/uploads/Lista-de-Repositorios-Recomendados_pt.pdf), preferencialmente com um identificador persistente como o digital object identifier (DOI). O acesso a tais dados com o link para o repositório deverá ser informada na seção “Disponibilidade de dados”. Deve haver citação dos dados no texto, preferencialmente nos “Métodos”, e a referência completa deve ser incluída na seção de “Referências”, seguindo exemplo: Andrade M. Estudo de genes em ratos albinos na América Latina. OSF [dataset], 2018, ASM0000v1. http://dx.doi.org/10.1590/0123-45620187214

Durante o processo editorial, editores podem solicitar o acesso às bases de dados do estudo aos autores para subsidiar a avaliação."

- Tabela 2: na primeira coluna, alinhar os dados a esquerda.

Solicitamos que, após os ajustes, seja enviada uma nova versão do manuscrito acompanhada de carta-resposta detalhada, indicando ponto a ponto como as observações foram incorporadas.

Recommendation: Minor revision

## Third round comments

Prezados autores,

Agradeço a submissão do manuscrito RESS-2025-0534.R2 intitulado “Tendência e carga de amputações de membros inferiores por diabetes: análise de série temporal, Amazonas, 2012-2021” à Revista Epidemiologia e Serviços de Saúde: revista do SUS (RESS).

Após a revisão, verificamos avanços relevantes em relação à versão anterior, entretanto, a seção de Aspectos éticos ainda necessita de ajustes. Trata-se de ajustes de revisão menor:

• Aspectos éticos: A recomendação anterior quanto à retirada da seção foi atendida, estando corretamente apresentada logo após o resumo. Contudo, conforme as Instruções aos autores da RESS, essa informação deve ser apresentada no formato de quadro, contendo obrigatoriamente: (i) o nome do comitê de ética em pesquisa que aprovou o estudo; (ii) o número do parecer; (iii) a data de aprovação; (iv) o número do certificado de apresentação para apreciação ética (CAAE); e (v) o registro do consentimento livre e esclarecido. Nos casos em que não houver apreciação ética, deve-se justificar sucintamente o motivo. Recomenda-se consultar exemplos de estudos já publicados pela RESS para assegurar a adequada padronização.

Recommendation: Minor revision

